# What have we learned from long-term studies in juvenile idiopathic arthritis? – Prediction, classification, transition.

**DOI:** 10.1186/s12969-025-01070-x

**Published:** 2025-02-19

**Authors:** Marite Rygg, Filipa Oliveira Ramos, Ellen Berit Nordal

**Affiliations:** 1 Department of Clinical and Molecular Medicine, Faculty of Medicine and Health Sciences (IKOM), Norwegian University of Science and Technology (NTNU), Trondheim, Norway; 2 Department of Pediatrics, St. Olavs University Hospital, Trondheim, Norway; 3 Pediatric Rheumatology Unit, Hospital Universitário ULS Santa Maria, Lisbon, Portugal; 4 Faculdade de Medicina, Universidade de Lisboa, Lisbon, Portugal; 5 Department of Clinical Medicine, The Arctic University of Norway (UiT), Tromsø, Norway; 6 Department of Pediatrics, University Hospital of North Norway (UNN), Tromsø, Norway

**Keywords:** Juvenile idiopathic arthritis, Adult, Outcome, Prediction, Classification, Transition

## Abstract

**Background:**

Research and management of juvenile idiopathic arthritis (JIA) are challenging due to its heterogeneous nature, chronicity, and unpredictable, multidimensional long-term outcomes.

**Main body:**

Long-term studies have consistently shown that a majority of children with JIA reach adulthood with ongoing disease activity, on medication, or with recurrent flares. The heterogeneity is evident both between and within the present JIA categories based on The International League of Associations for Rheumatology (ILAR) JIA classification system. Several baseline predicting factors are known, but prediction modelling is only in the initial phase, and more models need to be tested in independent cohorts and possibly also supplemented with new biomarkers. Many have criticized the ILAR classification system, but new or updated classification systems have not yet been validated and proved their superiority. The lack of prediction possibilities for long-term outcomes and the limited alignment between JIA classification categories and adult rheumatic conditions are challenges for research, may limit the accessibility to treatment, and hamper a smooth transition to adult care.

**Conclusion:**

We need more prospective, long-term studies based on unselected JIA cohorts with disease onset in the biologic era that can aid decision-making for individualized early treatment, suggest intervention studies, and ensure our patients the best possible transition to adulthood and the best likelihood of optimal health and quality of life.

## Background

Juvenile idiopathic arthritis (JIA) is one of the most common paediatric rheumatic disorders, but prognostication and classification are challenging due to heterogeneity both in clinical phenotypes and disease course [1, 2]. The International League of Associations for Rheumatology (ILAR) classification criteria divide JIA into seven categories based mainly on clinical characteristics during the first 6 months from disease onset [3]. This classification system is said to be a work-in-progress [4]. As the course and outcome differ markedly both within and between the ILAR categories, it is challenging to inform about the future course and choose the optimal medication for a child with new-onset JIA. A new or updated classification system has been advocated [5]. While some patients achieve remission during childhood, many continue to experience active disease or flares into adulthood, with varying degrees of disability and long-term consequences for physical and mental health, underscoring the importance of a smooth transition to adult care [6].

In the past, several long-term studies were performed to describe the outcome of JIA [7–14]. One of the challenges of these studies was the lack of validated outcome measures. The Wallace preliminary criteria and the American College of Rheumatology (ACR) provisional criteria for inactive disease and remission in JIA, have made the comparison of study results easier [15, 16]. In this paper, which is based on lectures held at the 31st annual European Paediatric Rheumatology Congress (PReS) Meeting in Gothenburg in September 2024, we will review recent long-term studies that follow children into adulthood, include the full spectrum of disease, and use validated outcome measures. Relevant papers and studies were selected by comprehensive literature searches in relevant databases by all three authors as preparation for their lectures, but no systematic search or analyses were performed.

## Outcome in adulthood

In Table 1, we have included some of the most important long-term studies that include proportion of participants in clinical remission off medication (CR). Most of these studies have used the preliminary Wallace criteria or the ACR provisional criteria for disease status [15, 16], but in some studies modifications of these criteria were used. The studies differ highly in design, from prospective population-based [17, 18] or hospital-based [19], to retrospective hospital-based [20], register-based [21], register-based biased to the most severe cases [22, 23], or to mixed design [24]. Even if they have all been published within the last decade, only the Research In Arthritis In Canadian Children Emphasizing Outcomes (ReACCh-Out) study [24] included only children diagnosed in the post-biologic era. Independent of the different designs, all but one study [19] demonstrated that less than half of the children (11–47%) were in remission off medication in adulthood. Even those who achieve symptom-free periods in childhood may experience disease flares as adults [25]. The one study demonstrating a slightly higher remission rate [19], had the longest observation time, 30 years, and used a slightly modified definition of remission where 69% were not re-examined at the final study visit. The rate of remission in this study might have been over-estimated.

**Table 1:** Patients in clinical remission off medication in adult juvenile idiopathic arthritis in long-term studies

Studies, country	*n*	Disease duration Years	Age at visit Years	CR^a^ %	Study design
Bertilsson, 2013 Sweden	86	17	n.a.	40^b^	Prospective, population-based Diagnosis 1984-86
Selvaag, 2016 South-East Norway	176	30	39	59^c^	Prospective, hospital-based Referral 1980-85
Oliveira-Ramos, 2016 Portugal	426	23	34	12	Cross-sectional, national register-based (“Reuma.pt register”), *n* = 355 retrospectively registered in adulthood
Dimopoulou, 2017 Greece	102 (302^d^)	17	25	24 (47^d^)	Retrospective, hospital-based (*n* = 102) + telephone interview of *n* = 205 lost-to follow-up. *N* = 74 diagnosed before 2000
Minden, 2019 Germany	701	14	23	11	Prospective register-based (“BiKeR/JUMBO registry”), selected to bDMARDs-treated individuals
Glerup, 2020 Nordic countries	434	18	24	33	Prospective, population-based (“Nordic JIA study”) Disease onset 1997–2000
Chhabra, 2020 Canada	247	6	17	47	Prospective, inception cohort (“ReACCH-Out cohort”) with retrospective long-term data collection. Diagnosis 2005–2020
Oliveira-Ramos, 2023 Portugal	361	20	29	15	Cross-sectional, national register-based (“Reuma.pt register”), selected to bDMARDs-treated individuals

*CR* clinical remission off medication, *Reuma.pt Register* Rheumatic Diseases Portuguese Registry, *BiKer/JUMBO* Biologika in der Kinderrheumatologie (BiKer) and Juvenile Arthritis Methotrexate/Biologics Long-Term Observation (JUMBO) registers, *bDMARDs* biologic diseasemodifying anti-rheumatic drugs, *JIA* juvenile idiopathic arthritis, *ReACCH-Out* Research in Arthritis in Canadian Children Emphasizing Outcomes inception cohort

^a^According to the Wallace 2004 and/or ACR provisiona criteria 2011 unless otherwise commented

^b^EULAR definition of remission: Inactive disease off medication for ≥ 2 years

^c^Including 69% assessed only by questionnaires (CR at 15-year follow-up and no history of flare assessed by questionnaire after 23 and 30 years)

^d^Including *n* = 205 lost to follow-up and assessed only by telephone interview

Compared to earlier studies, recent data show significant improvements in functional impairment, with severe disability reported in only 3–11% of adults with JIA [18, 26], although nearly half still experienced some degree of physical limitation (Table 2) [17–20, 22, 27]. The proportion with disability was lower, around 28–30%, in more unselected cohorts [18, 20]. A polyarticular disease course was associated with higher disability scores, even among those in clinical remission [21, 28]. Permanent sequelae or damage are less studied and more difficult to compare in different studies due to different measures used when assessing damage. After more than 18 years of disease, 20% still experienced some damage (Table 2), with the extent varying by JIA categories [18, 21]. Adults with polyarticular rheumatoid factor (RF) negative and psoriatic JIA from the Nordic JIA cohort had higher articular and extra-articular damage than other categories [18], while in the Rheumatic Diseases Portuguese Registry (Reuma.pt), adults with systemic-onset JIA, and RF positive and RF negative polyarthritis had higher articular damage scores [21].

**Table 2:** Proportion of adults with juvenile idiopathic arthritis with physical disability and damage in long-term studies

Studies, country	*n*	Disease duration Years	Age at visit Years	CR^a^ %	Disability HAQ > 0 %	Damage	
						JADI-A ≥ 1 %	JADI-E ≥ 1 %
Bertilsson, 2013, Sweden	86	17	n.a.	40^b^	46	n.a.	n.a.
Selvaag, 2016, South-East Norway	176	30	39	59^c^	45^d^	n.a.	n.a
Dimopoulou, 2017, Greece	102 (302^e^)	17	25	24 (47^e^)	47 (30^e^)	87	58
Minden, 2019, Germany	701	14	23	11	42	n.a.	n.a
Tollisen, 2019, South-East Norway	96	19	25	n.a.	46^f^	n.a	n.a
Glerup, 2020, Nordic countries	434	18	24	33	28	20	13

*CR* clinical remission off medication, *HAQ* health assessment questionnaire (range 0–3) by self-report, *JADI-A* juvenile arthritis damage index – articular (range 0–72), *JADI-E* JADI – extraarticular (range 0–18) scored by the physician

^a^According to the Wallace 2004 and/or ACR provisional criteria 2011 unless otherwise commented

^b^EULAR definition of remission: Inactive disease off medication for ≥ 2 years

^c^Including 69% assessed only by questionnaires (CR at 15-year follow-up and no history of flare assessed by questionnaire after 23 and 30 years)

^d^Among these, HAQ ≥ 0.5 (moderate to severe disability) = 25%

^e^Including *n* = 205 lost to follow-up and assessed only by telephone interview

^f^Among these, HAQ ≥ 1.5 (severe disability) = 3%

The chronic nature of JIA is known to affect overall quality of life, with chronic pain, limited mobility, and uveitis contributing to both physical and psychological impacts [29]. Several studies show that adults with JIA generally report poorer physical health, more pain, and more fatigue than their peers [27, 30–32]. Health outcomes differ according to JIA categories, and those with persistent oligoarticular JIA generally report a better quality of life [26, 28]. Increased mental health issues, such as anxiety and depression, have been described in adolescents and young adults with JIA [33, 34], although results are inconsistent [35]. Mental health can potentially affect physical functioning and quality of life, as well as adherence to medication and risk of disease flares.

The high number of individuals who are not in clinical remission or experience permanent damage, reduced physical or mental health in adulthood underscores the need for better prediction tools and further refinement in classification criteria aiming for better and more individualized treatment options early in the disease course and a smooth transition to adult care later.

## Prediction

The urge to predict the long-term outcome in JIA is more important as we get better, but also more expensive and potentially more harmful drugs, and a much larger armamentarium of drugs [36, 37]. We need to know how we can individualize treatment to target persistent inactive disease. The most ideal way to understand what happens in the future is to follow an unselected cohort of newly diagnosed children prospectively and longitudinally into adulthood.

### Baseline risk factors predicting unfavourable outcome in the Nordic JIA study

With a population-based design, the Nordic JIA cohort [38, 39] included all newly diagnosed children with disease onset in 1997–2000 from specific areas in Norway, Sweden, Finland, and Denmark. In total, 510 children with a median onset age of 5.5 years were included at a baseline visit and followed prospectively for 18 years [18, 40]. Persistent oligoarticular and systemic JIA had the best outcome compared to other categories after eight [40] and 18 years [18] (Table 3). That systemic JIA, as a group, has a good outcome, has also been shown in other population-based long-term studies [19, 28]. Human leukocyte antigen B27 (HLA-B27) associated features, characteristic of enthesitis-related arthritis (ERA), such as sacroiliitis, enthesitis, and hip arthritis, were shown to be risk factors for unfavourable long-term outcomes [18, 41]. Likewise, psoriasis and psoriasis-associated features, as well as ankle arthritis within the first year after onset, were risk factors for not achieving remission [42–44]. Finally, self-reported pain six months after disease onset, predicted less remission, more physical disability, more persistent pain, and for the oligoarticular group; an increased risk for developing extended disease [45].

**Table 3:** Baseline factors predicting unfavourable adult outcome in long-term studies in juvenile idiopathic arthritis

Studies	*n*	Dis. Dur. Years^a^	Age Years^a^	Baseline factors	Long-term outcome
**The Nordic JIA study**					
Glerup, 2020	434	18	24	Categories other than persistent oligoarticular and systemic JIA, ERA	Not in remission off medication
Arnstad, 2021	377	18	23	Female sex, diagnostic delay	Fatigue
Rypdal, 2021	434	18	24	Early onset uveitis, ANA	Ocular complications
Glerup, 2024	236	18	24	Combined baseline factors^b^ with or without 16 baseline biomarkers	Inactive disease, remission off medication
Berntson, 2013	399	8	15	HLA-B27, sacroiliitis, enthesitis, hip arthritis	Not in remission off medication
Esbjörnsson, 2015	399	8	15	Ankle arthritis first year	Not in remission off medication
Ekelund, 2017	427	8	15	Psoriasis, psoriasis-associated features	Not in remission off medication
Nordal, 2017	435	8	15	Young age at onset, antihistone antibodies, ANA	Uveitis
Rypdal, 2018	423	8	15	Combined baseline characteristics (multivariable prediction model)	Not in remission, functional disability, joint damage (JADI-A > 0)
Arnstad, 2019	243	8	15	Pain intensity at baseline	Not in remission off medication, pain > 0, CHAQ/HAQ > 0, JADI > 0
**Other studies**					
Bertilsson, 2013	86	17	n.a.	None	Remission (EULAR definition), HAQ > 0, SF-36 PCS
Selvaag, 2016	176	30	39	HLA-DRB1*1	Active disease or on medication
Oliveira Ramos, 2016	426	23	34	Young age at disease onset, RF positive polyarticular and systemic JIA, ACPA positive	Active disease, higher HAQ, JADI-A or JADI-E
Dimopoulou, 2017	102	17	25	Polyarticular JIA	“Persistent disease”; proportion of time spent in active disease
Minden, 2019	701	14	23	n.a.	Not in “drug-free remission”, HAQ > 0
Tollisen, 2019	96	19	25	Pain, number of active joints, CHAQ ≥ 1	Physical disability (HAQ > 0), pain, physical HRQOL (SF-12 PCS)

*Dis. Dur.* disease duration, *JIA* juvenile idiopathic arthritis, *ERA* enthesitis-related arthritis, *ANA* antinuclear antibodies, *HLA-B27* human leukocyte antigen B27, *JADI-A* juvenile arthritis damage index – articular, *JADI-E* JADI – extraarticular, *CHAQ* child health assessment questionnaire (range 0–3), *HAQ* health assessment questionnaire (range 0–3), *EULAR* European league against rheumatism, *SF-36* medical outcome study Short Form based on 36 questions, *PCS* physical component summary score, *RF* rheumatoid factor, *ACPA* anticitrullinated protein antibodies, *n.a.* not assessed, *HRQOL* health-related quality of life, *SF-12* medical outcome study Short Form based on 12 questions

^a^Mean or median

^b^Gender, age at onset, active and cumulative joints, ESR and CRP

In addition to non-achievement of remission, other unfavourable outcomes have been studied. Anti-type II collagen antibodies, anti-cyclic citrullinated peptides (anti-CCP), and RF were risk factors for joint damage after 8 years [46]. Female sex and diagnostic delay predicted fatigue after 18 years [32]. Early disease onset in girls and positive antinuclear antibodies (ANA) in both sexes, were risk factors for developing uveitis during the disease course [47]. On the other hand, except for uveitis, no association to other outcomes was found for either ANA or onset age [32, 40, 48].

### Baseline risk factors predicting unfavourable outcome in other studies

Other long-term studies have also searched for baseline risk factors predicting unfavourable outcome (Table 3). Bertilsson et al. did not find any baseline features associated with long-term outcome [17]. In one of the studies with the longest follow-up ever, Selvaag et al. found that HLA-DRB1*1 predicted active disease 30 years after disease onset [19]. This is one of the few genetic markers, except HLA-B27, that have been associated with unfavourable long-term outcome. In the Portuguese Reuma.pt registry, young age at disease onset, RF positive polyarthritis, systemic JIA, and anti-CCP were predictors of unfavourable outcome [21]. The contradictory result regarding systemic JIA as a predictor of disease outcome in the Portuguese study compared to what was found in the Nordic JIA study [18], may reflect the different designs of the two studies. The prospective population-based design in the Nordic JIA cohort, ensured that all cases of systemic JIA were kept in the cohort, while in the Portuguese cross-sectional cohort, children in remission would not have been found as adults in the registry [21]. In a study from South-East Norway, early pain reports, number of active joints, and physical disability were found to predict unfavourable patient-reported outcomes [27], consistent with results from the Nordic JIA study showing that early pain report predicted fatigue in adulthood [32].

### Disease course factors predicting unfavourable long-term outcome

Disease course factors have also been shown to predict outcome in several long-term studies in JIA (Table 4). Both Bertilsson et al. [17] and Selvaag et al. [19] found that unfavourable outcome after 5 or 15 years of disease, predicted unfavourable outcome 17 or 30 years after disease onset. In a Greek study, longer duration spent in active disease the first 5 years predicted persistent disease, radiographic damage, and physical disability after 17 years [20]. In the Nordic JIA study, pain, self-reported poor health, active disease, and previous or ongoing disease-modifying anti-rheumatic drugs (DMARDs) at the 8-year visit predicted severe fatigue after 18 years [32]. Finally, results from both the German Biologika in der Kinderrheumatologie (BiKer), the Juvenile Arthritis Methotrexate/Biologics Long-Term Observation (JUMBO) registries [22], and the national Portuguese Reuma.pt registry [23] demonstrated that a late start of biologic DMARDs (bDMARDs), predicted unfavourable outcome compared to an early bDMARD start. None of these disease course factors can be used to select children in need of early aggressive treatment, but they all strengthen the hypothesis of a “window of opportunity” early in the disease course, where we possibly may change, or at least influence, the outcome [22, 49].

**Table 4:** Disease course factors predicting unfavourable adult outcome in long-term studies in juvenile idiopathic arthritis

Studies	*n*	Dis. Dur. Years^a^	Age Years^a^	Disease course factors	Long-term outcome
Bertilsson, 2013	86	17	n.a.	Unfavourable outcome at 5-year	Not in remission, HAQ > 0, SF-36 PCS
Selvaag, 2016	176	30	39	Unfavourable outcome at 15-year	Active disease or remission on medication
Dimopoulou, 2017	102	17	25	Longer duration of active disease first 5 years	Long duration in active disease during 17 years, radiographic damage
Arnstad, 2021	377	18	23	Unfavourable outcome at 8-year	Fatigue
Minden, 2019	701	14	23	Late start bDMARDs (> 2 years or > 5 years from onset)	Less drug-free remission, higher cJADAS10, higher HAQ, higher PatGA, cJADAS71 > 4.5
Oliveira Ramos, 2023	361	20	29	Late start of bDMARDs (> 5 years from onset)	Less remission off medication, higher HAQ, higher SF-36 PCS

*Dis. Dur.* disease duration, *n.a.* not assessed, *HAQ* health assessment questionnaire (range 0–3), *SF-36* medical outcome study Short Form based on 36 questions, *PCS* physical component summary score, *bDMARDs* biologic disease-modifying anti-rheumatic drugs, *cJADAS-10* clinical Juvenile Arthritis Disease Activity Score based on 10 joints (range 0–10), *PatGA* patient global assessment of disease impact on wellbeing (range 0–10), *cJADAS71* cJADAS based on 71 joints (range 0-101)

^a^Mean or median

### Prediction models in long-term studies

Most long-term studies focusing on prediction have studied single risk factors and associations to unfavourable outcome. In 2017 and 2018, two different models for individual prediction in JIA were developed [48, 50]. Based on the Canadian ReACCH-Out cohort, Guzman and co-workers developed a model for prediction of a severe disease course [50], while Rypdal and co-workers developed prediction models combining easily available baseline factors that gave acceptable sensitivity and specificity to predict unfavourable outcomes for individual participants in the Nordic JIA cohort [48]. The Nordic prediction models were later tested for external validation in the Canadian ReACCH-Out cohort with acceptable results [51], and likewise, the Canadian prediction model was tested in the Nordic cohort with excellent results [52]. Recently, two other prediction models were compared on a group basis in the Nordic JIA cohort: Model 1, including clinical baseline characteristics, erythrocyte sedimentation rate (ESR) and C-reactive protein (CRP), and model 2, using the same factors in addition to 16 baseline biomarkers, including the S100 proteins [53]. The latter model with biomarkers, performed significantly better in predicting disease status after 18 years. However, no internal or external validation of these latter models has presently been performed.

### Lessons learned about prediction

Long-term studies can give us important information on outcome in JIA. The strength of these studies is their ability to describe real-life adult outcome with all its multi-faceted dimensions, to define factors associated with different outcomes, to use these factors in prediction modelling, and finally, to obtain hypotheses for future intervention studies. An important limitation is that today’s outcome studies will always reflect yesterday’s treatment practices. When using data from long-term studies, we are studying the effect of old practices. Also, a multitude of different factors during the disease course may influence the outcome independently of robust baseline predictors. Lastly, a model can never be better than the factors you add into it, and knowledge of which factors that are of main importance and how they interact is still limited.

## Classification

The heterogeneity of JIA calls for long-term studies to identify meaningful sub-entities, both for research and clinical purposes. There are two main challenges in classification research, where the first is to include the whole spectrum from mild to severe JIA. We must avoid selection bias with overestimation of the severe polyarticular and systemic categories mostly followed in tertiary centres where research initiatives often originate. The second challenge is to capture the longitudinal course, because the cross-sectional application of any criteria will be too simplistic and underestimate the chronicity of the disease [54]. Thus, challenges in JIA research are to perform population-based studies or at least as unselected studies as possible, with a longitudinal design and a long-term perspective.

### Challenges with the ILAR classification criteria

The ILAR classification criteria defining seven categories were the first to unify previous diverging terminology used in different parts of the world, such as juvenile rheumatic arthritis (JRA) and juvenile chronic arthritis (JCA) [3]. Recent emerging evidence from genetic, pathophysiological, and clinical studies question the current validity in several aspects:

1. An arbitrary division between polyarthritis and oligoarthritis, the latter involving a maximum of four joints during the first six months of disease [55].
2. Different terminology for similar diseases among children and adults that hampers transition [21, 56].
3. An arbitrary upper age limit of 16 instead of 18 years, which is the most common age for transition to adult care in most chronic diseases [56, 57].
4. The absolute criterion of arthritis in the definition of systemic JIA, which may delay start of targeted medication [55, 58].
5. Too stringent exclusion criteria potentially excluding children with psoriatic arthritis and ERA from new targeted medications [42, 43, 59].

### Alternative JIA classification systems

The Paediatric Rheumatology INternational Trials Organisation (PRINTO) preliminary classification criteria were published as a new alternative by Martini and coworkers in 2019 [55]. The *early-onset ANA positive* group is described as a separate childhood-specific disorder with a high risk of developing asymptomatic uveitis. Four disorders partly overlap with the ILAR categories; *systemic JIA*, *RF positive JIA*, *enthesitis-/spondylitis-related JIA*, and finally *undefined* and *other JIA disorders*, where the first three have adult arthritis equivalents. While *undefined JIA* fulfils criteria for more than one of the above groups, *other JIA* does not fulfil the criteria of any of the defined groups and is stated to be more clearly defined later. Notably, the terminology for psoriasis in combination with arthritis is still discussed and not yet settled [60].

There are other ongoing initiatives, such as the classification work based on the Canada-Netherlands Personalized Medicine Network in Childhood Arthritis and Rheumatic Diseases (UCAN) inception cohort [61], presently comprising over 2100 JIA patients. Five clusters are identified based on demographic and clinical features, joint patterns, and new biologic markers such as cytokines, interferons, and proinflammatory genes; Juvenile axial spondyloarthritis, juvenile peripheral spondyloarthritis, juvenile rheumatoid arthritis, juvenile oligoarthritis, and noteworthy, one of the clusters also according to this classification, is the early onset arthritis.

### Longitudinal changes in ILAR categories

Changes in ILAR categories over time have been found in 33% of the Nordic JIA cohort from six months after disease onset to assessments after eight and 18 years [40, 62]. While the systemic and polyarticular RF positive JIA categories remained stable, 84 of 230 individuals with oligoarticular arthritis at baseline developed extended disease, and only 113 of 230 remained persistently oligoarticular after 18 years [62]. In the baseline polyarticular RF negative category, 17% changed to psoriatic, ERA, or undifferentiated arthritis. The ERA category increased, and psoriatic arthritis more than tripled in size after 18 years. The undifferentiated category also increased, in contrast to the intended role as a temporary category expected to decrease over time as disease determinants evolved. The ILAR exclusion criteria drive ERA and psoriatic arthritis towards the undifferentiated category, and altogether 43% of children with psoriasis were not categorized with psoriatic arthritis [42].

### Similarities between ILAR categories and adult rheumatic conditions

In the JIA Immunochip consortium, polyarticular RF positive JIA resembled adult seropositive rheumatoid arthritis (RA) in genetic profile, while polyarticular RF negative and oligoarticular JIA resembled seronegative RA [63, 64]. This result is in line with a study based on the Portuguese Reuma.pt register comparing ILAR *versus* adult criteria in 426 adult individuals with JIA [21]. They concluded that the systemic, RF positive polyarthritis, and psoriatic arthritis categories showed more than 90% overlap with adult conditions. Altogether, 21% were non-classifiable, with persistent and extended oligoarthritis having the highest proportion of non-classifiable cases.

### Comparison of JIA classification systems

Comparison of the ILAR to the PRINTO preliminary JIA classification criteria was performed in two large prospective cohorts; the Canadian ReACCH-Out cohort and the British Childhood Arthritis Prospective Study (CAPS) cohort [65, 66]. Using the ILAR classification system, the undifferentiated arthritis group constituted 12% of the ReACCH-Out cohort and 21% of the CAPS cohort. Using the PRINTO preliminary classification system, 63% of the ReACCH-Out cohort and 70% of the CAPS cohort were classified as *other JIA disorders*. *Early onset ANA positive JIA* constituted 20% in both cohorts. Both studies concluded that the PRINTO preliminary classification system defined a large proportion of JIA patients as *other JIA disorders* in need of further characterization.

In the Canadian ReACCH-Out cohort, 389 patients were also classifiable according to adult rheumatic conditions and then compared with the corresponding JIA groups [65]. Altogether, 15% of the individuals with adult conditions were assigned to undifferentiated arthritis according to the ILAR classification system, while 56% were assigned to *other JIA disorders* according to the PRINTO preliminary classification system. The conclusion was that the adult rheumatic conditions aligned better with ILAR categories than with PRINTO disorders in the present preliminary version.

### Lessons learned about classification from long-term studies

Several arguments are in favour of a new or modified JIA classification system. Studies in adults and children show significant overlap between anti-CCP and RF and support the inclusion of anti-CCP in addition to or as an alternative to RF in the classification of polyarticular JIA [46, 55, 67]. The exclusion criteria in the ILAR classification system have been a challenge, especially for defining ERA and psoriatic arthritis [42]. Removing the arbitrary cut-off for affected joints between oligoarticular and polyarticular RF negative JIA, seems to be timely. Improving alignment with adult rheumatic diseases will not only simplify paediatric drug trials and therefore increase access to treatment, but may also enable a smoother transition process especially if the age of onset in JIA is extended to 18 years [21, 56]. In the future, new biomarkers incorporated in the JIA classification system, might enable identification of more homogeneous categories paving the road to more mechanism-directed treatments [68].

A drawback with a new JIA classification system, is the fact that biomarker-based clustering using machine-learning techniques has shown partly diverging results [61]. Additionally, JIA research based on ILAR categories over the past 30 years will be difficult to compare to new studies. Lastly, it is essential that a new set of classification criteria must show validity and feasibility in cohorts from different geographic areas and ethnicities before worldwide adoption.

## Transition

Even if the physical health and functioning of adolescents with JIA have improved during the last decades, long-term studies have shown that many, if not most, will reach adulthood with a chronic condition and challenges that need attention from specialized care. The transition process for adolescents with JIA is essential, not merely as a transfer of care to adult rheumatology but as a step towards more autonomy in the health management for adolescents. Transition should be a structured and planned process starting at the beginning of adolescence, with the aim to assess medical, psychosocial, and educational needs to give the adolescents the necessary skills to be gradually more independent in management of all aspects related to their health [69].

Inadequate transition from paediatric to adult care is associated with loss of follow-up, increased risk of stopping treatment, more flares, and increased disability due to poorly controlled arthritis or uveitis [70]. Young patients are particularly vulnerable as they are prone to risky behaviours that can jeopardize the control of their condition and lead to worsening of their disease [71]. To minimize this risk, several position statements have been developed to guide healthcare providers to give the best care while transitioning patients [72–74]. The most effective and pragmatic transition programs are built based on key components that help to guide successful transitions. Structured programs generally have a transition coordinator who is responsible for managing the transition process and communication between paediatric and adult teams [75]. Joint clinics, where paediatric and adult rheumatologists co-manage patients across the transition period, have been found to improve continuity of care [76]. Educating the adolescents to improve their knowledge of their disease and treatment options, is also a key factor [77]. Best practices may also include the use of readiness assessment tools to assess self-management skills gaps and to individualize transition plans specific to each patient [78, 79]. Integrated psychosocial support and peer mentoring programs have been demonstrated to positively impact key health outcomes, as well as improve patient satisfaction and facilitate engagement during the transitional phase [80].

There is not a single best model for a transition program. Programs must be adapted to the resources available in each centre. However, the multidisciplinary co-management of the patient, shared or, at least, connected medical record databases, and coordinated communication between paediatric and adult services are crucial to prevent long-term adverse outcomes, as well as to monitor drug safety and efficacy [81].

There are several obstacles to the success of transitional care for adolescents with JIA, including absence of validated tools to assess disease activity in adulthood, lack of specific treatment guidelines for adults with JIA, absence of adolescent-specific training of adult rheumatologists, and shortage of resources [69, 81, 82]. Another important issue to address, which is not covered in current recommendations, is how to select patients who need to be transferred to an adult unit when they leave paediatric care [25]. Some of these issues could be difficult to overcome but are essential to improve the transition programs and guarantee quality of life, satisfaction with care, and better long-term outcomes.

### Lessons learned about transition

Transitioning from paediatric to adult care can be challenging due to variability in disease progression and outcomes across JIA categories. This highlights the need for a structured, multidisciplinary approach to the transition process that should begin early in adolescence. There is a significant risk of loss to follow-up in patients lacking robust transitional support, together with an increased risk of flares and disability. To overcome these challenges, it is essential to standardize transition pathways with effective communication between paediatric and adult rheumatologists and specific training for transitioning patients. It also calls for strong and flexible transition models that address the patient population and characteristics of each centre. Evidence suggests that enhancing transitional care relates not only to the successful management of JIA but also to these patients’ overall future health and quality of life.

## Conclusion

JIA management remains challenging due to its heterogeneous nature and long-term health consequences. To predict the outcome of our patients better than today, we need more studies from the post-biologic era, preferably prospective and population-based, including baseline clinical characteristics, independent of ILAR categories that may change. We will probably need baseline biomarkers and maybe also baseline imaging markers that have not been a focus in this paper, as well as validated outcome measures. We will need international collaboration for testing the models in different populations and finally intervention studies for confirmation of the predictive ability of the models. In our opinion, a revision of the JIA classification system is clearly needed. As for now, a limited revision may be the best option because new classification criteria for JIA must prove to be clearly superior to the ILAR classification system and better aligned with adult conditions. Long-term studies emphasize the need for holistic transition programs that consider not only medical management but also psychosocial and educational well-being to facilitate smooth integration into adult care, enabling people with JIA to enjoy as healthy adult lives as possible.

## Data Availability

No datasets were generated or analysed during the current study.

## Abbreviations

ACR: The American College of Rheumatology
anti-CCP: Anti–cyclic citrullinated peptides
ANA: Antinuclear antibodies
bDMARDs: Biologic disease-modifying anti–rheumatic drugs
Biker: Biologika in der Kinderrheumatologie (BiKer)
CAPS: The Childhood Arthritis Prospective Study
CHAQ: Child Health Assessment Questionnaire
CR: Clinical remission off medication
CRP: C–reactive protein
DMARDs: Disease–modifying anti–rheumatic drugs
ERA: Enthesitis–related arthritis
ESR: Erythrocyte sedimentation rate
HLA: Human leukocyte antigen
ILAR: The International League of Associations for Rheumatology
JCA: Juvenile chronic arthritis
JIA: Juvenile idiopathic arthritis
JUMBO: Juvenile Arthritis Methotrexate/Biologics Long–Term Observation
JRA: Juvenile rheumatic arthritis
PReS: European Paediatric Rheumatology Congress
PRINTO: The Paediatric Rheumatology INternational Trials Organisation
RA: Rheumatoid arthritis
ReACCh-Out: The Research In Arthritis In Canadian Children Emphasizing Outcomes
Reuma.pt: The Rheumatic Diseases Portuguese Registry
RF: Rheumatoid factor
UCAN: Canada–Netherlands Personalized Medicine Network in Childhood Arthritis and Rheumatic Diseases
